# CAFOs: Thorne Responds

**DOI:** 10.1289/ehp.10124R

**Published:** 2007-07

**Authors:** Peter S. Thorne

**Affiliations:** Environmental Health Sciences Research Center, The University of Iowa, Iowa City, Iowa, E-mail: peter-thorne@uiowa.edu

I am pleased that the National Pork Board has read the Mini-Monograph on Environmental Health Impacts of Concentrated Animal Feeding Operations (CAFOs) (Bunton et al. 2007; Burkholder et al. 2007; Donham et al. 2007; Gilchrist et al. 2007; Heederik et al. 2007; Thorne et al. 2007); I hope the board has given consideration to the 26 recommended priority research needs and 16 recommendations for translating science to policy. These were carefully developed by the 31 authors in the five workgroups on Airborne Exposures, Monitoring and Modeling, Water Quality, Infectious Disease Epidemics and Antibiotic Resistance, and Community Health.

Wagstrom states her belief that the process of developing the reports was flawed; because she was not involved in the process, she likely does not know what the process was. In 2002, I convened a group of seven professors, each with decades of experience with this issue and representing environmental health, exposure analysis, occupational medicine, veterinary medicine, environmental policy, water quality, and infectious diseases. We considered existing environmental health problems associated with the industrialization of agriculture and developed goals and plans for the conference. In 2003 we submitted a grant proposal to the National Institutes of Health and, based on extramural peer-review, received a grant to host the conference. We conferred widely with experts in the field to identify those scientists actively engaged in research relevant to the conference topics. We then invited recognized scientists with peer-reviewed publications on the subjects of the workgroups to speak at the plenary session and participate in the ensuing workgroups. In addition, three state regulators provided expertise in exposure modeling. The workgroups considered the problems and formulated a set of research and policy needs that were presented to the full workshop. Based on feedback, these presentations were further developed by the workgroups into articles that were peer-reviewed by *EHP* before publication in the Mini-Monograph. Multiple levels of peer review are the cornerstone of modern science, and I believe this is the best process to develop science to support environmental health policy.

Wagstrom states that the papers fail to “differentiate between potential risks associated with general animal production—regardless of facility type—and risks associated with CAFOs.” As stated in the overview to the Mini-Monograph (Thorne 2007), this conference was focused on industrialized livestock production because this is how > 85% of the pork and poultry in the United States and western Europe is produced. Of U.S. swine production, 54% occurs in 110 industrialized facilities, each housing > 50,000 hogs, and 78.5% occurs in operations with > 5,000 animals (U.S. Department of Agriculture 2007).

Wagstrom states that “a true assessment of potential risk requires an assessment of exposure” and that this “has not been addressed to any extent in these reports.” This comment is particularly perplexing because each of the articles addressed exposure assessment issues, and two of the reports dealt almost exclusively with exposure assessment, monitoring and modeling of exposures, harmonization of exposure assessment methods, mixed exposures from CAFOs, and establishment of monitoring networks for characterization of exposures. Wagstrom’s suggestion that we should ignore lagoon breaches or manure pipe ruptures with regard to water quality and focus on daily operations is misguided because these events occur with regularity and lead to significant surface water contamination, fish kills, and loss of recreational use of surface waters.

The report of the Workgroup on Infectious Disease Epidemics and Antibiotic Resistance was given a particularly large task, given that there are > 1,500 scientific papers indexed in PubMed (National Library of Medicine 2007) each year on avian influenza and 230/year on antibiotic resistance in livestock. It was not their task to provide a survey of livestock zoonoses.

Wagstrom also raises questions about the Danish experience after the antibiotic growth promotant (AGP) ban. The facts are that consumption of antibiotics for livestock production in Denmark was > 200 metric tons in 1994, 160 tons in 1997 when antibiotics were last used as growth promotants for weanling and finishing pigs, and dropped to 114 tons in 2005 when this use had ceased (Table 1) [Danish Integrated Antimicrobial Resistance Monitoring and Research Program (DANMAP) 2006]. Pig production increased over this same period from 21 to 26 million hogs (DANMAP 2006). Pig mortality during the last 10 years increased from 6% to 8.2%, with some of the increase attributable to the AGP-ban but much of the increase due to a new viral infectious disease (postweaning multisystemic wasting syndrome) that arose in 2000 (Dahl J, personal communication).

This Mini-Monograph represents current, peer-reviewed science and recommendations developed by leading independent researchers in the field. We welcome further dialog with the National Pork Board and other producer groups so that the United States can achieve needed environmental health improvements in the livestock industry.

## Figures and Tables

**Table 1:** Danish use of antibiotics for livestock production.

Year	Antibiotic use (kg)	Swine produced
1994	207,000	21,000,000
1997	160,000	22,000,000
2005	114,000	26,000,000

Data from DANMAP (2006).
